# Photovoltaic Grid-Connected Modeling and Characterization Based on Experimental Results

**DOI:** 10.1371/journal.pone.0152766

**Published:** 2016-04-01

**Authors:** Ali M. Humada, Mojgan Hojabri, Mohd Herwan Bin Sulaiman, Hussein M. Hamada, Mushtaq N. Ahmed

**Affiliations:** 1 Faculty of Electrical & Electronics Engineering, University Malaysia Pahang, Pekan, Malaysia; 2 Electricity Production Directorate of Salahaldeen, Ministry of Electricity, 34007, Baiji, Iraq; 3 Faculty of Civil Engineering & Earth Resources, Universiti Malaysia Pahang, 26300, Gambang, Malaysia; Chongqing University, CHINA

## Abstract

A grid-connected photovoltaic (PV) system operates under fluctuated weather condition has been modeled and characterized based on specific test bed. A mathematical model of a small-scale PV system has been developed mainly for residential usage, and the potential results have been simulated. The proposed PV model based on three PV parameters, which are the photocurrent, I_*L*_, the reverse diode saturation current, *I*_*o*_, the ideality factor of diode, *n*. Accuracy of the proposed model and its parameters evaluated based on different benchmarks. The results showed that the proposed model fitting the experimental results with high accuracy compare to the other models, as well as the I-V characteristic curve. The results of this study can be considered valuable in terms of the installation of a grid-connected PV system in fluctuated climatic conditions.

## 1 Introduction

Solar energy is an important energy source that can be harvested from nature free of cost. The earth’s surface receives hundreds of times more energy from the sun than the worldwide demand [1]. The collection of this energy and its conversion into a more useful source such as electrical power for daily usage is important; nevertheless, technologies have their own boundaries and difficulties that must be resolved before photovoltaic (PV) implementation on a large scale. Of these difficulties, PV system modeling and characterization and the performance compared to other conventional sources are the most important [2, 3].

Precise PV modeling has posed a challenge for researchers in order to achieve a model that strictly emulates the characteristics of a PV system within specified or non-specified climatic conditions. Unfortunately, PV modeling and characterization are location dependent, and every researcher developed a model on the basis of the available location and climate at a specific place [4]. In addition, the site of PV installation might affect the output power and eventually the PV performance in a positive or negative way.

Different previous studies have been reported for the power systems modeling. Some of the studies focused on comparative models [5–7], based on either equivalent circuit type [5, 7], or the system type [6]. The multi-swarm particle swarm optimization method, was employed by Hu et al., [5] to find the optimal model parameters, which used twelve equivalent circuit models for Li-ion batteries, selected from state-of-the-art lumped models stated in the previous works. Hu et al., [6], also examined three different system types from a Li-ion battery pack, a super capacitor pack, and a dual buffer, for a hybrid bus. In another study, three different types of equivalent circuit models for ultra-capacitors were tested comparatively [7]; the classic model, the multi-stage ladder model and the dynamic model, which support in improving the battery charging status [8]. Another study characterized the dynamic model by fitting the collected impedance data [9], to further verify the reliability of the model used. Meanwhile, an online model identification of lithium-ion battery for electric vehicles implemented [10]. The model presented a reasonable level of complexity, whereas the validation results showed that the on-line calibrated model can accurately predict the dynamic voltage behavior of the lithium-ion battery. However, in [11, 12] a prolonged Kalman Filter was used to recursively estimate the model parameters, where in [11] equivalent circuit model template composed of a bulk capacitor, a second-order capacitance-resistance network, and a series resistance. While an efficient model [12] structured from a second-order resistance-capacitance network and a simple analytical open circuit voltage, versus state of charge (SOC) map was applied to characterize the voltage performance of a lithium iron phosphate battery for electric vehicles.

In terms of PV modeling, many studies have been carried out with PV system modeling and characterization [13–19]. According to [13], an approach for finding the parameters of a single-diode model of a PV panel was proposed. The influence of both irradiation and temperature on these parameters was considered. However, methods for finding the model parameters in the case of instantaneous variations in the temperature and irradiance have not been considered. In [15], a PV model was built upon simple procedures to acquire the PV system parameters in an easy and fast manner. This model was used mainly to characterize these parameters by using MATLAB/ Simulink. Nonetheless, the applicability of using this model is limited because the model did not consider fast climate change. In 2014, an improved four-parameter model was constructed [17]. Therefore, this study was conducted to build a model that is able to fit the variation in the current—voltage characteristics (I–V) under various operating conditions. However, the lack of a practical analysis in this study made the validation weak. The improved modeling technique in [17] is based on genetic-algorithm optimization with a number of PV panel parameters determined using this approach. Two models were developed: a single-diode model and double-diode model. On the basis of this study, the single-diode model is more efficient than the double-diode model. Nevertheless, it has computational steps in the computational process.

In this paper, we introduce a novel method presents a reliable and an accessible PV model distinct in accuracy of model parameters and its characteristics based on changing of both ambient temperature and irradiance level. Therefore, the current model differs from other compared models by accuracy and also flexibility of application under fluctuated weather, while the other models just based on specific weather conditions rather than another. Another contribution introduced in this study, the I-V and P-V characteristics measurements to ensure reliable results of the model parameters. Finally, this study provides valuable information for those who are interested in PV system modeling and installation in the fluctuated weather conditions.

## 2 PV System Modeling and Characterization

In this section, the methodology of PV system characterization is presented in detail. A five-parameter model is used to characterize the PV array. An accurate mathematical model for the PV system is derived on the basis of the realistic parameters derived from the mathematical calculations.

### 2.1 PV Array Modeling

**Fig 1:**
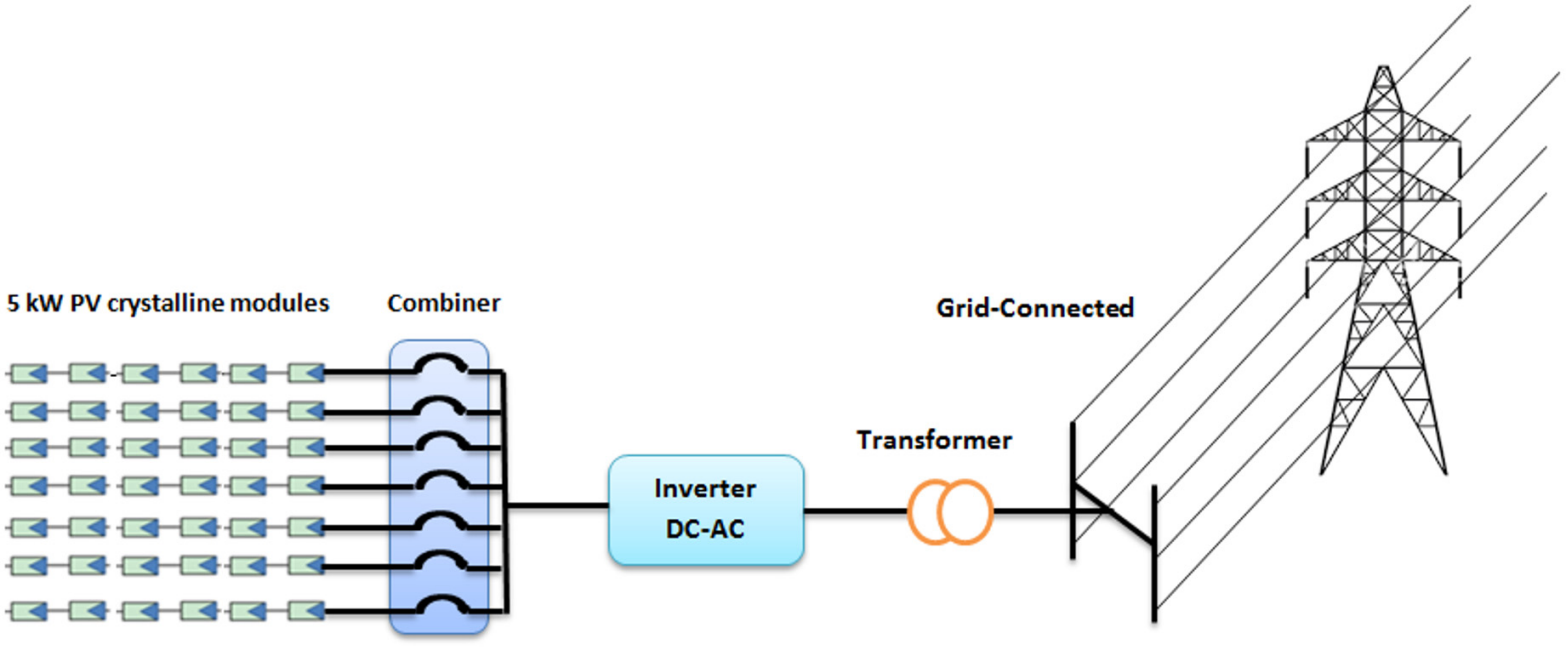
Schematic diagram of the PV system installed under Malaysia weather condition.

In this study, a 5 kW PV system with 42 multicrystalline PV modules installed in Malaysia is consider as a case study, as shown in Fig 1. The system specifications are listed in Table 1.

**Table 1:** Specifications of the proposed system.

Parameter	Value and Units
Number of PV modules	42
Maximum Power (P_MP_)	120 W
Maximum Voltage (V_MP_)	16.9 V
Maximum Current (I_MP_)	7.10 A
Open-Circuit Voltage (V_OC_)	21.5 V
Short-Circuit Current (I_SC_)	7.45 A

In general, the PV system in this study is modeled on the basis of a single-diode model. Thus, a solar cell can be modeled by an equivalent circuit that consists of a current source, a reverse-biased diode, and a resistor connected in series, where each part of the model is associated with a specific parameter. The cell’s photocurrent is represented by a current source, shown as the solar cell in the model. Moreover, the output voltage is represented by the reverse-biased diode. The diode’s saturation current is represented by a shunt resistance. Finally, the cell’s internal losses are indicated by a series resistance.

Under normal operation conditions or in the absence of sunlight, PV cell performance will be similar to a semiconductor diode in the dark. In such a case, this PV cell is represented by a normal diode, where its characteristics (current—voltage) can be expressed as

$$I_{D}=I_{o}\left(.\;exp^{\frac{q.V_{D}}{nkT}}-1\right)$$ (1)

and

$$V_{D}=V+IR_{S},$$ (2)

where *I* is the PV output current, *I*_*o*_ is the reverse saturation current of the diode, *q* is the electron charge equal to 1.602×10^−19^ C, *k* is the Boltzmann constant (1.381×10^−23^ J/K), *T* is the ambient temperature, *V*_*D*_ is the voltage across the diode terminals, *V* is the output voltage, *R*_*S*_ is the series resistance, and *n* is the ideality factor, also known as the quality factor or sometimes the emission coefficient.

On the other hand, an ideal cell is represented as a current generator connected in parallel with a diode when the solar cell is illuminated, and its I–V characteristics are described by Shockley [22] with the following equation:

$$I=I_{L}-I_{D}=I_{L}-I_{o}\left(exp{\;}^{\frac{q.\left(V+IR_{S}\right)}{nkT}}-1\right)-\frac{V+IR_{s}}{R_{sh}}.$$ (3)

*n* usually takes values in the range 1–2 (although it might be larger in very few situations), which depends on the construction and semiconductor material.

Generally, the proposed PV system model is a five-parameter model, namely, *I*_*L*_, *I*_*o*_, *a*, *R*_*s*_, and *R*_*sh*_. This model can be used for an individual cell, a module consisting of several cells, or an array consisting of several modules.

Nonetheless, owing to the difficulty in finding an accurate value for *n*, we assume that

$$C_{1}=\frac{q}{nk}.$$ (4)

Then,

$$a=\frac{T_{c}}{C_{1}}.$$ (5)

As a result of this new relation as well as the expected higher shunt resistance, the shunt current becomes modest, and eventually, the new I–V characteristic equation can be simplified as

$$I=I_{L}-I_{o}\left(exp{\;}^{\frac{\left(V+IR_{S}\right)}{a}}-1\right),$$ (6)

$$V=a.\text{ ln}\left(\frac{I_{L}-I}{I_{o}}\right)-IR_{S}.$$ (7)

Usually, the photocurrent (*I*_*L*_) is proportional to the value of the solar irradiation, and it is supposed to be linearly dependent on the effective cell temperature (*T*_*c*_). Consequently, this light-generation current could be expressed as

$$I_{L}=\left(C_{2}+C_{3}.T-C_{4}\right)G_{T},$$ (8)

where C_*2*_–C_*4*_ are coefficients, *G*_*T*_ is the solar irradiance, and *T* is the temperature of the cell. In the related literature [8], researchers demonstrated that *T*_*c*_ affects the diode saturation linearly as

$$I_{o}=C_{5}.T^{3}.\text{exp}\left(-\frac{C_{6}}{T}\right),$$ (9)

where C_*5*_ and C_*6*_ are coefficients.

### 2.2 Solar Inverter Characterization

The performance of a PV system depends on more than one factor such as the inverter performance; this section is dedicated to a discussion of the inverter’s characteristics. The inverter used in this study is Sunny Mini Central SMC 6000 inverter, which is the closest match to practical implementations.

In fact, a direct current/alternating current (DC/AC) inverter converts the DC current produced by the PV array into AC current. The efficiency of this energy conversion process can be expressed by

$$\eta \left(t\right)=\left(P_{in}\left(t\right)-P_{loss}\left(t\right)\right)/P_{in}\left(t\right),$$ (10)

where *P*_*in*_ is the input power from the PV array, and *P*_*loss*_ is the conversion power loss.

In addition, a general relation between the inverter efficiency and the PV power is implemented in order to model and characterize the inverter as

$$\eta_{inv}=A\;P_{pv}^{n}+B\text{ for }0\;<\;P_{pv}\;<\;N,$$ (11)

$$\eta_{inv}=AP_{pv}+B\text{ for }P_{pv}\;\geq \;N,$$ (12)

where *P*_*pv*_ is the PV power, and *A*, *B*, and *N* are constants.

Therefore, the method used in this study models an inverter on the basis of a correlation method between the curve (characteristic curve) provided by the manufacturer and the experimental results. Thus, it may be an efficient model, which should be applicable for all inverter types in any climatic conditions.

Generally, Eqs (11) and (12) are applicable to this type of inverter (Sunny Mini Central inverter) in terms of the inverter efficiency. However, they can also be used and applied to other inverters of this kind. Therefore, the user needs to derive new equations for other types of inverters to obtain a more accurate inverter model.

## 3 Model Evaluation Criteria

An efficient evaluation between the real curves of the photovoltaic panels and the proposed models has been done in this study. This evaluation is based on many benchmarks to ensure the accuracy of the proposed model with respect to the real data. All of these criteria show that the proposed model has a margin of error with respect to the real data from the PV panels. An evaluation based on the most famous factors such as the mean absolute percentage error (MAPE), the sum of the squares due to the error (SSE), the sum of squares due to regression (SSR), the sum of squares due to the total (SST), and the SSR divided by the SST (R-squared) is executed in this study. All of these factors are expressed as

$$MAPE=\left\{\left|\frac{\left(R_{v}-P_{m}\right)}{Rv}\right|/n\right\}*100\%,$$ (13)

$$SSE=\Sigma_{i=1}^{n}{\left(Rv_{i}-Pm_{i}\right)}^{2},$$ (14)

$$SSR=\Sigma_{i=1}^{n}{\left(Pm_{i}-Cel_{i}\right)}^{2},$$ (15)

SST=SSR+SSE, (16)

$$R^{2}=SSR/SST,$$ (17)

where *Rv* is the real value, *Pm* is the proposed model value, *n* is the number of tested points, and *Cel* is the central horizontal line drawn horizontally from the center of the theoretical curve.

## 4 Results and Discussion

In this work, experimental data collected under Malaysian climatic conditions to obtain accurate values for the constants (*C*_1_–*C*_6_) of the global PV model. As mentioned in Table 1, the selected system consists of a 5 kW grid-connected PV system, which has been investigated by simulation for a month (from May 1, 2014 to May 31, 2014). Throughout this period, the irradiations ambient temperature, PV output power, and inverter efficiency are recorded every 0.1 s.

*C*_1_–*C*_6_ are accurately estimated on the basis of the experimental results in Table 2.

**Table 2:** Values of *C*_1_–*C*_6_.

Coefficient	Value
*C*_1_	0.0062
*C*_2_	0.0048
*C*_3_	6.745×10^−6^
*C*_4_	3.237×10^−8^
*C*_5_	2.677×10^−4^
*C*_6_	6399

This work has focused on *a*, *I*_*L*_, and *I*_*o*_. In other works such as [5], the characteristics are based on only *a* and *I*_*L*_. Additionally, in other research works [6–8], the model only depends on the equations for *I*_*L*_ and *I*_*o*_. Furthermore, the current work targets the new parameters within the tropical site (Malaysia as the case study), which might be applicable for all tropical areas for the first time. A set of new equations based on the extracted values are as follows:

$$a=\frac{T_{c}}{0.0062},$$ (18)

$$I_{L}=\left(0.0048+6.745\text{E}-6.T-0.025\right)G_{T},$$ (19)

$$I_{o}=2.677\text{E }-4.T^{3}.\text{exp}\left(-\frac{6399}{T}\right).$$ (20)

**Fig 2:**
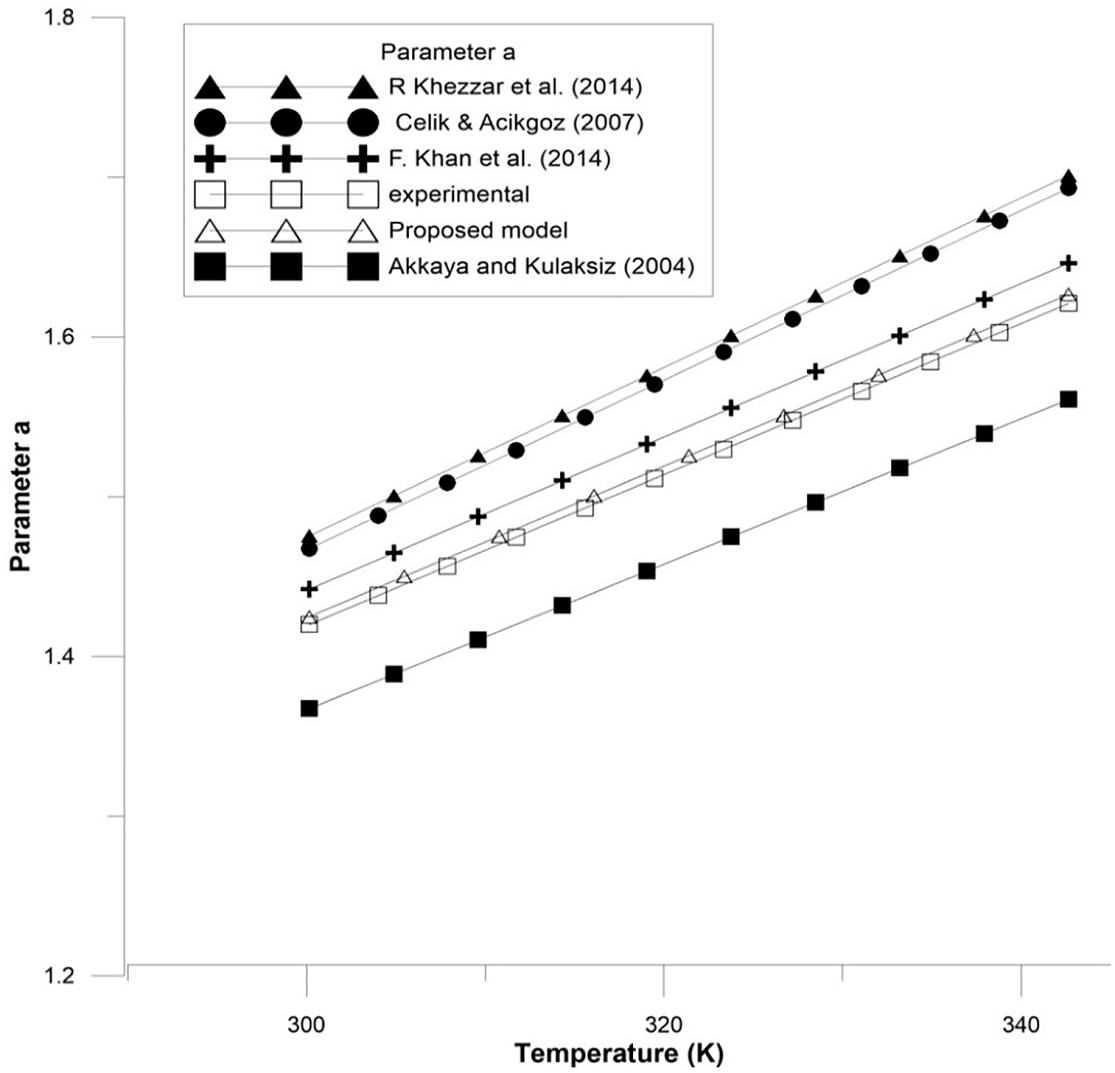
A comparison between the proposed equation for the parameter *a* and the experimental results with other models.

**Fig 3:**
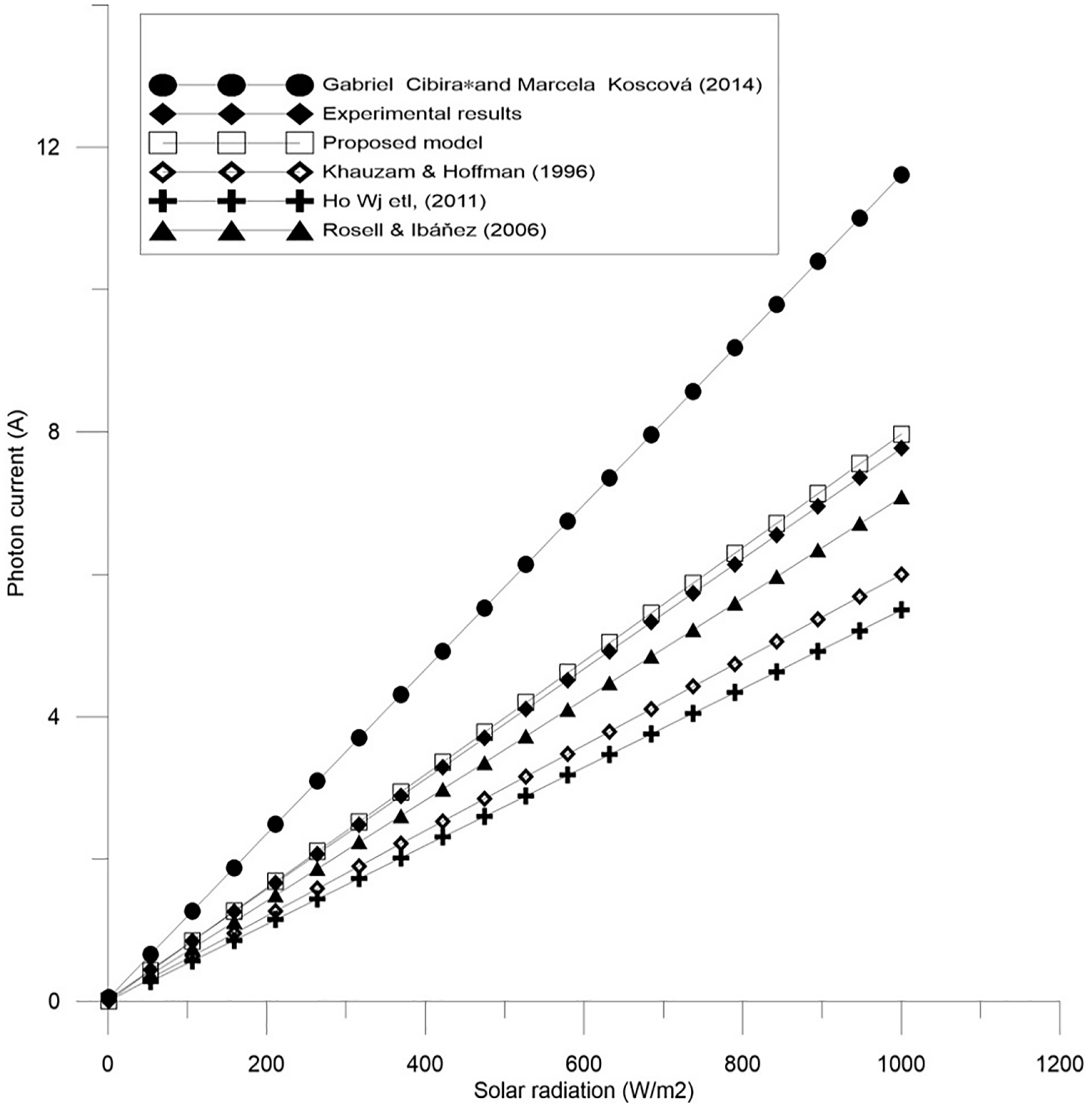
A comparison between the proposed equation for the photocurrent, *I*_*L*_ and the experimental results with other models.

**Fig 4:**
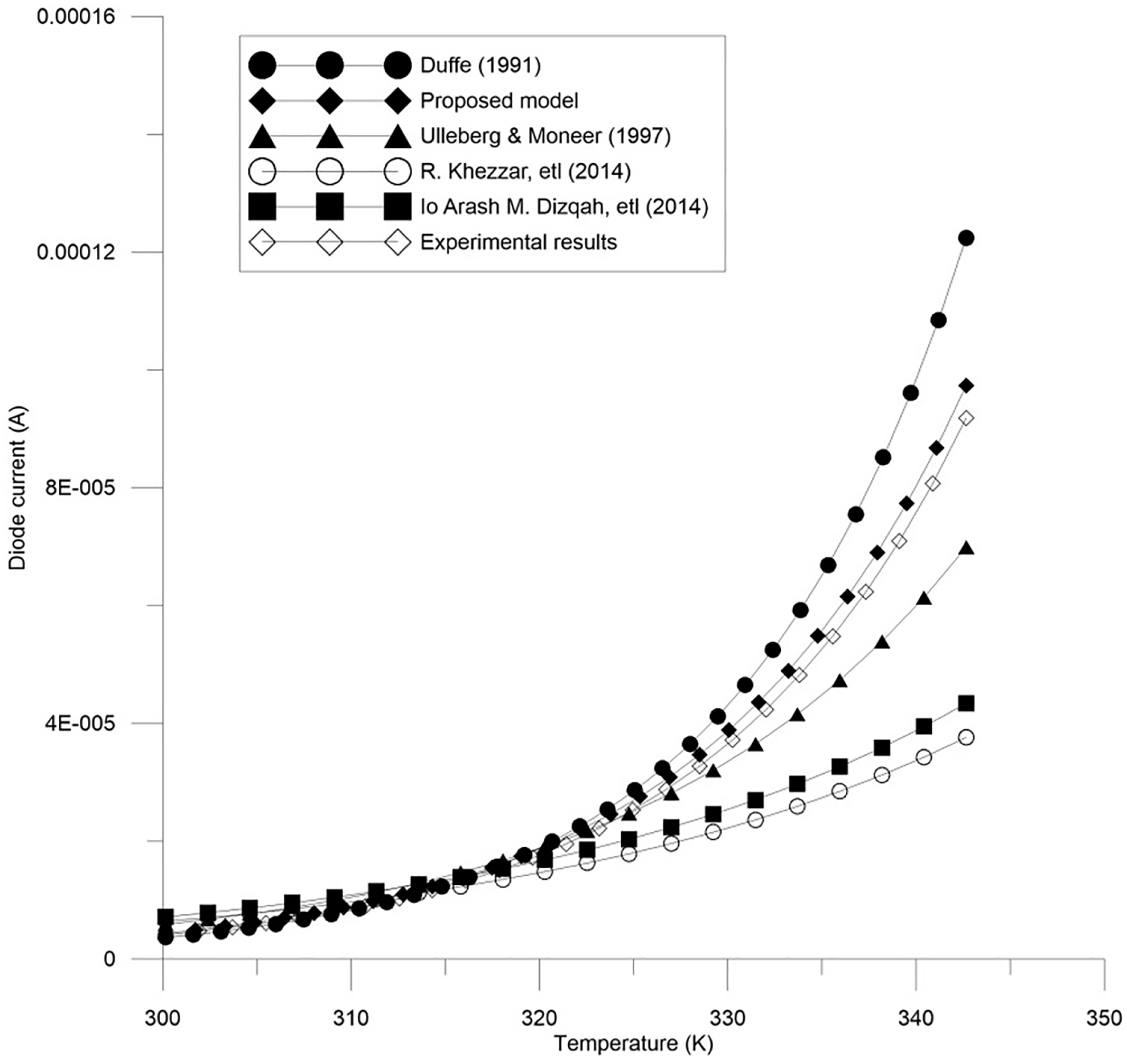
A comparison between the proposed equation for the reverse diode current, *I*_*o*_ and the experimental results with other models.

Figs 2–4 show the results for parameters *a*, *I*_*L*_, and *I*_*o*_.

Generally, a validation of this study is utilized to confirm the accuracy of the proposed model, where it is based on several benchmarks such as the MAPE, R-squared, SSE, SSR, and SST, as stated before. The model evaluation criteria are presented in Table 3.

**Table 3:** Evaluation criteria of the proposed model.

Evaluation Criteria	Parameter *a*	Parameter *I*_*o*_	Parameter *I*_*L*_
MAPE	3.12%	6%	10.3%
SSE	2.198×10^−16^	2.7281×10^−15^	7.216×10^−14^
R-squared	0.9998	0.998	0.997
SSR	1.09878×10^−12^	1.36132×10^−12^	2.39812×10^−11^
SST	1.099×10^−12^	1.364×10^−12^	2.405×10^−11^

Although the PV model depends on five parameters (*I*_*o*_, *I*_*L*_, *a*, *R*_*s*_, and *R*_*sh*_), [20–23] some studies have focused on four parameters [24] or three parameters (*R*_*s*_, *R*_*sh*_, and *a*) [25]. However, in this research, *R*_*sh*_ is ignored because *I*_*sh*_ is too small. *R*_*s*_ is assumed to be fixed [3] and equal to 4.85 mΩ. Thus, the experimental data for the other three parameters (*I*_*L*_, *I*_*o*_, *a*) are used to estimate an accurate and exact value for *C*_1_–*C*_6_ by comparing them with the theoretical results. Nevertheless, owing to the rapid and continuous fluctuations in the weather conditions such as the radiance and temperature, continuous data for the current and voltage (every 0.5 s) have been logged.

**Fig 5:**
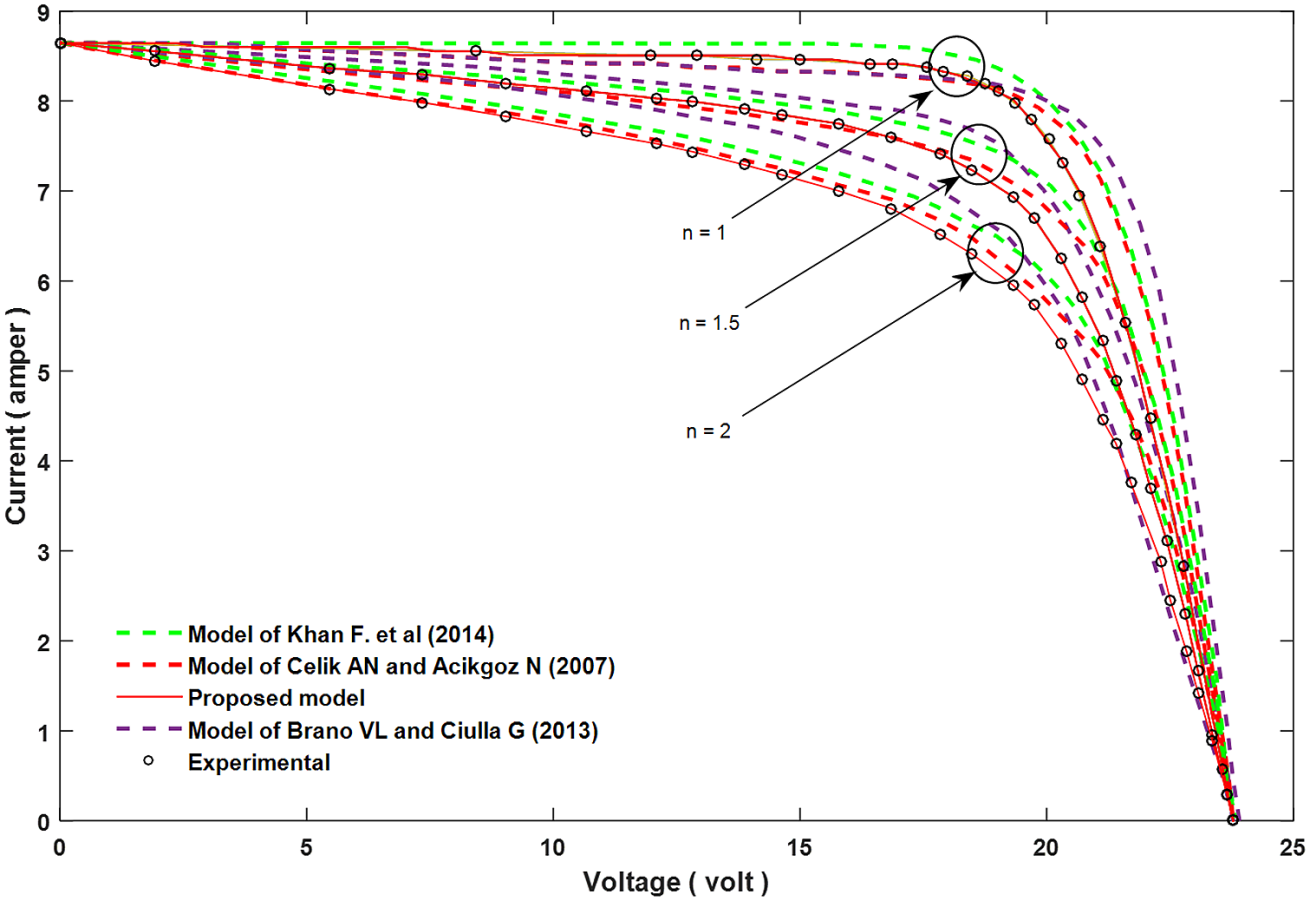
Influence of changing parameter “*a”* on the I–V characteristic curve, and comparing with experimental results and other models.

**Fig 6:**
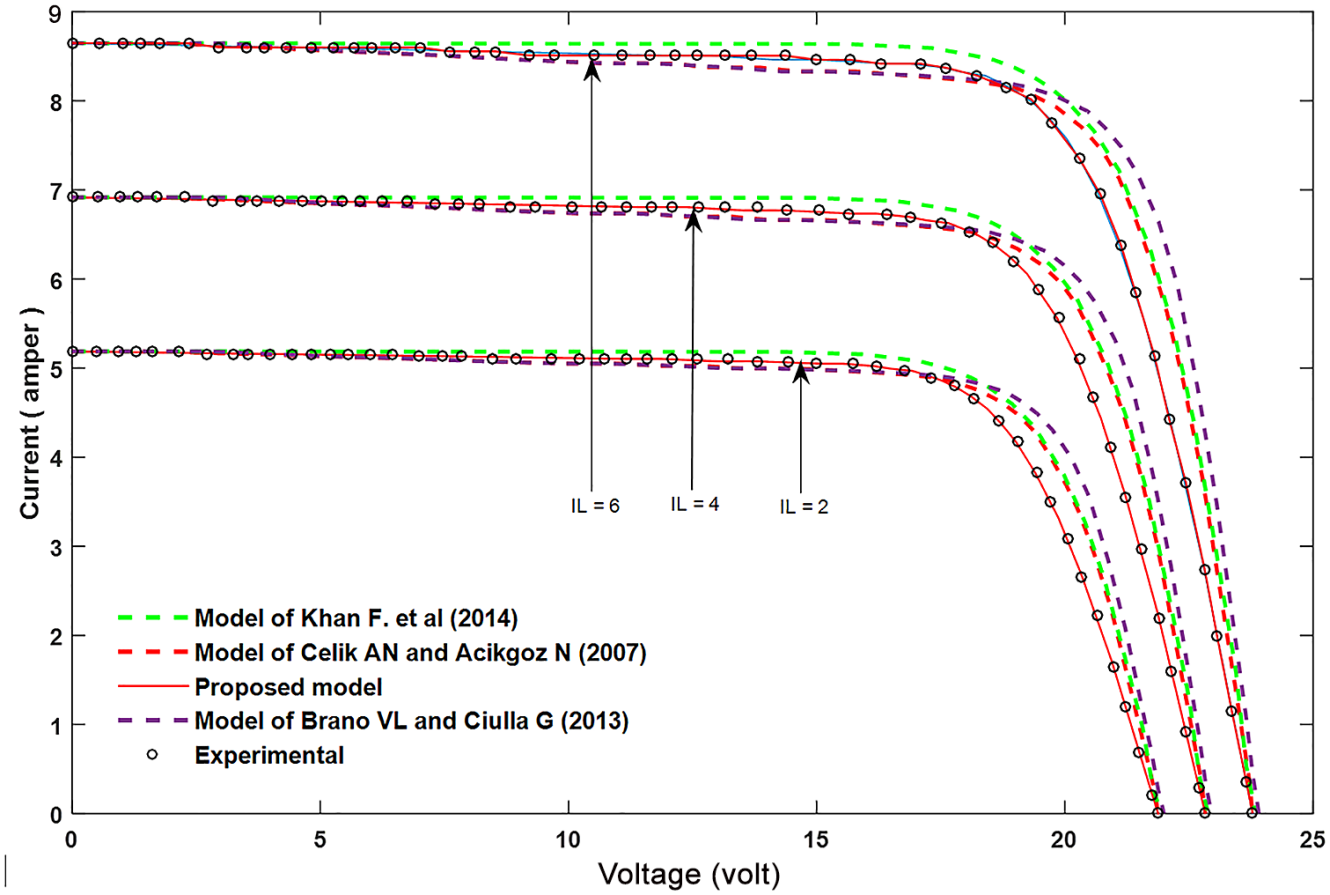
Influence of changing *I*_*L*_ on the I–V characteristic curve, and comparing with experimental results and other models.

**Fig 7:**
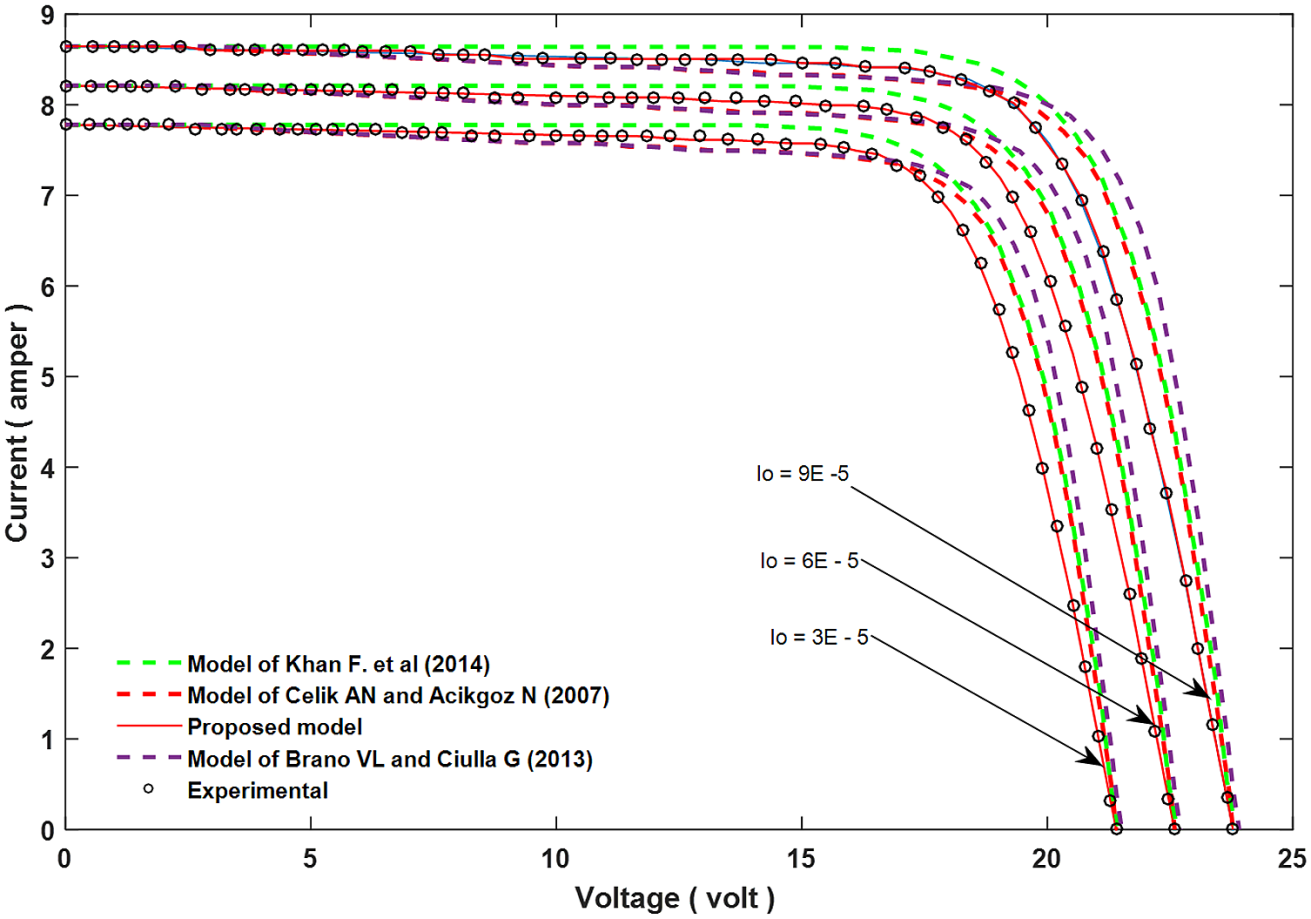
Influence of changing *I*_*o*_ on the I–V characteristic curve, and comparing with experimental results and other models.

In order to confirm the accuracy of the proposed model, a comparison is conducted between the real values (experimental results), the proposed theoretical values, and other models done before, for all of the parameter *a*, *I*_*L*_, and *I*_*o*_ which are shown in Figs 5–7. Furthermore, these figures also present the performance of these parameters and its effect on the I-V and P-V characteristics.

The characterization results of number of meteorological datasets are collected on the basis of a month-long data, from sunrise to sunset. The MAPE, SSE, R-squared, SSR, and SST were employed to verify the accuracy of the model. The predicted values at each data point were compared with the measured values, providing the accuracy of the model. For instant, the least-squares error (SEE) for this study is close to zero, which indicates a good performance of the proposed mode. In addition, the R-squared values generally exhibited a good indication of the coefficient values used for this model, which are close to 1. Based on the above mentioned evaluation criteria, which used in the model parameters validation, the results of evaluation is listed in Table 3.

**Fig 8:**
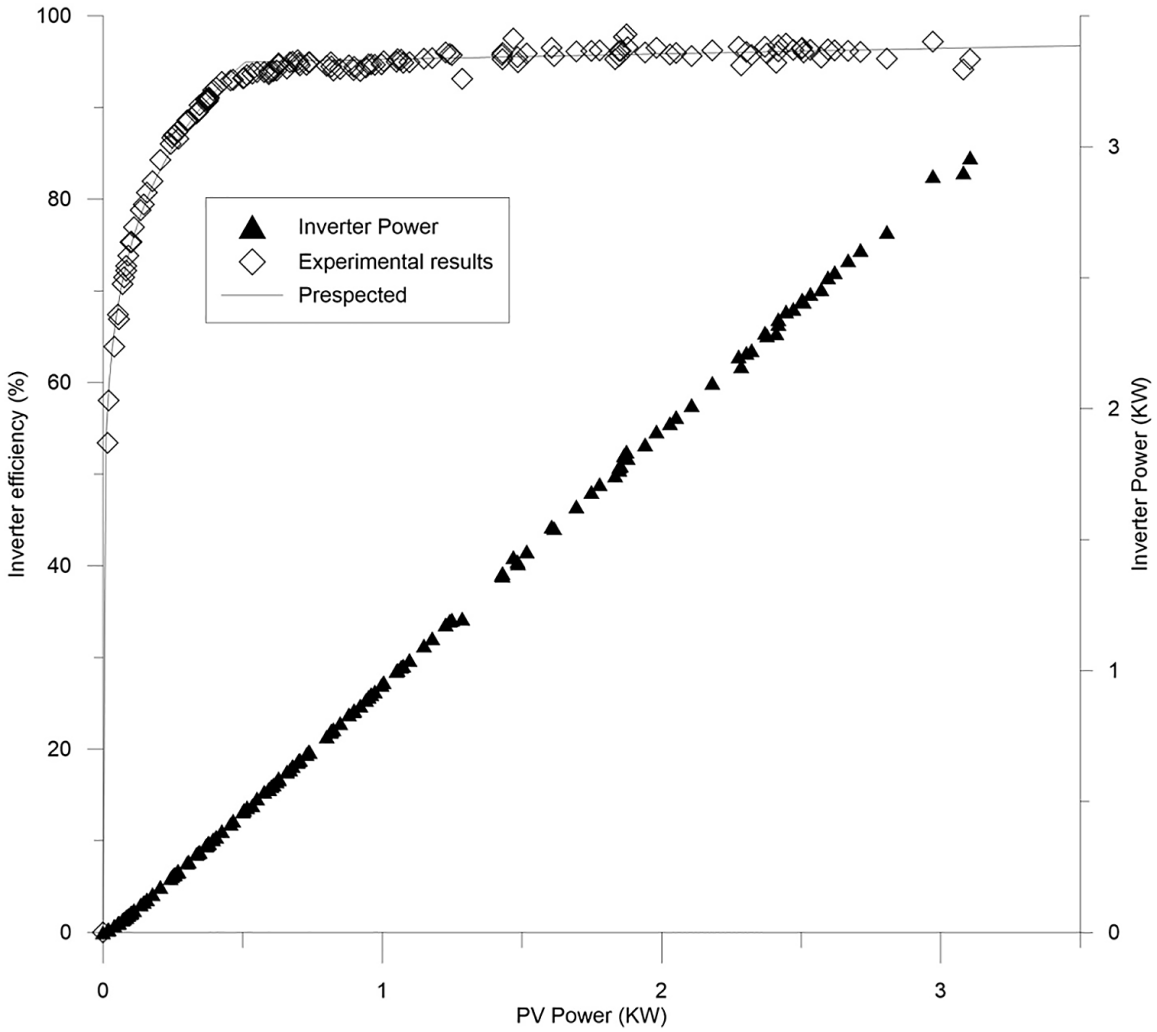
Inverter’s power and efficiency with respect to PV power.

However, with respect to inverter characterization an innovative way is used in this study to derive an accurate relation between inverter efficiency and the PV power, which is based on a correlation method. This method tries to correlate the real values of the curve with the curve provided by the manufacturer. Eventually, a new relation has been derived from this fitting as the standard for all inverter efficiency of this kind. The accuracy of this method was tested through finding MAPE to be only1.09%, and R-square to be 0.99998, proving its high degree of accuracy of this type of inverter. The evaluation for the inverter’s efficiency which has been executed through the expected and real results is presented in Fig 8, and the relation is represented by equations below.

$$\eta_{inv}=105.1\;P_{pv}^{0.1456}\text{ for }0\;<\;P_{pv}\;<\;0.517$$ (21)

$$\eta_{inv}=0.6032P_{pv}+94.64\text{ for }P_{pv}\;\geq \;0.517$$ (22)

### Conclusion

Experimental and theoretical calculations have been performed to determine the PV model’ parameters and I-V characteristic for a grid-connected photovoltaic system which is operated under fluctuated weather condition. In this purpose, an accurate mathematical PV model has been accomplished through theoretical and experimental evaluation of the PV parameters (the photocurrent, I_*L*_, the reverse diode saturation current, *I*_*o*_, the ideality factor of diode, *n*). Results of these parameters showed that the proposed model verified valued accuracy (based on different evaluation criteria) comparing with the other models. Moreover, the results of I-V characteristics based on changing all of the used model parameters also fitted the real results and recorded effect of each parameter on the I-V characteristics alone, which didn’t deal before in the literature.

## Supporting Information

S1 Table Experimental data of PV characteristics. The main PV model parameters were derived based on real data for a PV system. Due to the huge amount of these data, in this study just attached a sample for the first day from May 2014. The table below illustrate these dependent sample data. Each date sample is taken for an average of five minute time.
(DOCX)
