# Supplementary material for: Photovoltaic Grid-Connected Modeling and Characterization Based on Experimental Results
Source: PLoS One. 2016 Apr 1;11(4):e0152766. doi: 10.1371/journal.pone.0152766 (PMC4817980; doi:10.1371/journal.pone.0152766)
Supplement: S1 Table — The main PV model parameters were derived based on real data for a PV system. Due to the huge amount of these data, in this study just attached a sample for the first day from May 2014. The table below illustrate these dependent sample data. Each date sample is taken for an average of five minute time. (DOCX) [file pone.0152766.s001.docx]

**S1 Table, experimental data of PV characteristics**

| Date | PYRANOMETER | PVTemp | Amb.Temp. | Iac | Vac | Ipv | Vpv |
| --- | --- | --- | --- | --- | --- | --- | --- |
|  | W/m2 | °C | °C | A | V | A | V |
|  |  |  |  |  |  |  |  |
| 5/1/2014 7:15:00 | 25.08 | 28.30 | 24.81 | 0.000 | 0.00 | 0.000 | 0 |
| 5/1/2014 7:20:00 | 25.08 | 28.28 | 24.75 | 0.000 | 0.00 | 0.000 | 0 |
| 5/1/2014 7:25:00 | 25.08 | 28.17 | 24.84 | 0.035 | 0.02 | 0.055 | 0.024 |
| 5/1/2014 7:30:00 | 23.82 | 28.18 | 24.89 | 0.120 | 0.15 | 0.175 | 0.163 |
| 5/1/2014 7:35:00 | 25.08 | 28.19 | 24.93 | 0.201 | 0.17 | 0.266 | 0.187 |
| 5/1/2014 7:40:00 | 25.08 | 28.33 | 25.16 | 0.266 | 0.32 | 0.334 | 0.35 |
| 5/1/2014 7:45:00 | 26.17 | 28.47 | 25.36 | 0.298 | 0.56 | 0.354 | 0.612 |
| 5/1/2014 7:50:00 | 31.35 | 28.68 | 25.45 | 0.379 | 0.81 | 0.433 | 0.875 |
| 5/1/2014 7:55:00 | 31.05 | 28.80 | 25.48 | 0.404 | 1.05 | 0.444 | 1.137 |
| 5/1/2014 8:00:00 | 33.10 | 28.88 | 25.57 | 0.518 | 1.29 | 0.553 | 1.4 |
| 5/1/2014 8:05:00 | 43.89 | 29.14 | 25.85 | 0.613 | 1.53 | 0.627 | 1.663 |
| 5/1/2014 8:10:00 | 49.01 | 29.45 | 26.18 | 0.794 | 1.77 | 0.783 | 1.925 |
| 5/1/2014 8:15:00 | 50.15 | 29.79 | 26.49 | 0.984 | 2.01 | 0.941 | 2.188 |
| 5/1/2014 8:20:00 | 54.33 | 30.29 | 26.82 | 1.158 | 2.25 | 1.098 | 2.45 |
| 5/1/2014 8:25:00 | 68.96 | 30.85 | 27.10 | 1.279 | 2.50 | 1.228 | 2.713 |
| 5/1/2014 8:30:00 | 75.23 | 31.45 | 27.71 | 1.368 | 2.74 | 1.302 | 2.976 |
| 5/1/2014 8:35:00 | 91.95 | 31.79 | 27.86 | 1.405 | 2.98 | 1.353 | 3.238 |
| 5/1/2014 8:40:00 | 100.31 | 32.20 | 28.05 | 1.491 | 3.22 | 1.431 | 3.501 |
| 5/1/2014 8:45:00 | 118.84 | 33.22 | 28.44 | 1.809 | 3.46 | 1.615 | 3.763 |
| 5/1/2014 8:50:00 | 149.46 | 35.53 | 28.50 | 1.501 | 3.56 | 1.355 | 3.867 |
| 5/1/2014 8:55:00 | 147.04 | 36.22 | 28.67 | 1.346 | 3.65 | 1.249 | 3.97 |
| 5/1/2014 9:00:00 | 125.39 | 35.28 | 28.58 | 1.322 | 3.75 | 1.293 | 4.073 |
| 5/1/2014 9:05:00 | 139.43 | 34.97 | 28.96 | 1.607 | 3.84 | 1.481 | 4.176 |
| 5/1/2014 9:10:00 | 145.45 | 35.30 | 30.11 | 1.674 | 3.94 | 1.515 | 4.28 |
| 5/1/2014 9:15:00 | 145.90 | 35.45 | 29.93 | 1.951 | 4.03 | 1.742 | 4.383 |
| 5/1/2014 9:20:00 | 142.83 | 36.58 | 29.73 | 2.127 | 4.13 | 1.889 | 4.486 |
| 5/1/2014 9:25:00 | 121.97 | 37.90 | 30.34 | 1.513 | 4.22 | 1.431 | 4.589 |
| 5/1/2014 9:30:00 | 200.62 | 39.49 | 31.15 | 2.475 | 4.32 | 2.319 | 4.693 |
| 5/1/2014 9:35:00 | 221.51 | 41.92 | 32.67 | 2.857 | 4.41 | 2.595 | 4.796 |
| 5/1/2014 9:40:00 | 224.69 | 43.78 | 33.28 | 3.155 | 4.51 | 2.862 | 4.899 |
| 5/1/2014 9:45:00 | 218.73 | 45.14 | 33.20 | 3.013 | 4.60 | 2.726 | 5.002 |
| 5/1/2014 9:50:00 | 185.35 | 44.46 | 32.03 | 2.544 | 4.70 | 2.319 | 5.106 |
| 5/1/2014 9:55:00 | 156.43 | 43.51 | 31.70 | 2.338 | 4.79 | 2.146 | 5.209 |
| 5/1/2014 10:00:00 | 150.46 | 43.43 | 31.99 | 2.304 | 4.89 | 2.113 | 5.312 |
| 5/1/2014 10:05:00 | 176.68 | 43.42 | 31.87 | 2.592 | 4.98 | 2.389 | 5.415 |
| 5/1/2014 10:10:00 | 176.59 | 43.88 | 31.95 | 2.636 | 5.08 | 2.467 | 5.519 |
| 5/1/2014 10:15:00 | 189.12 | 43.90 | 31.77 | 2.873 | 5.17 | 2.635 | 5.622 |
| 5/1/2014 10:20:00 | 187.42 | 43.55 | 32.02 | 2.920 | 5.27 | 2.729 | 5.725 |
| 5/1/2014 10:25:00 | 180.99 | 43.52 | 32.28 | 2.945 | 5.36 | 2.687 | 5.828 |
| 5/1/2014 10:30:00 | 202.62 | 44.12 | 32.80 | 3.307 | 5.46 | 2.987 | 5.932 |
| 5/1/2014 10:35:00 | 219.43 | 44.54 | 32.60 | 3.520 | 5.55 | 3.142 | 6.035 |
| 5/1/2014 10:40:00 | 226.83 | 44.84 | 33.32 | 3.772 | 5.65 | 3.373 | 6.138 |
| 5/1/2014 10:45:00 | 243.46 | 46.03 | 33.86 | 3.977 | 5.74 | 3.570 | 6.241 |
| 5/1/2014 10:50:00 | 277.10 | 46.71 | 33.73 | 4.358 | 5.84 | 4.057 | 6.345 |
| 5/1/2014 10:55:00 | 242.79 | 47.31 | 34.01 | 3.925 | 5.93 | 3.683 | 6.448 |
| 5/1/2014 11:00:00 | 223.60 | 47.80 | 33.41 | 3.620 | 6.03 | 3.324 | 6.551 |
| 5/1/2014 11:05:00 | 225.69 | 47.85 | 33.83 | 3.785 | 6.12 | 3.409 | 6.654 |
| 5/1/2014 11:10:00 | 227.88 | 48.15 | 34.83 | 3.863 | 6.22 | 3.498 | 6.758 |
| 5/1/2014 11:15:00 | 232.53 | 48.17 | 34.48 | 3.881 | 6.31 | 3.577 | 6.861 |
| 5/1/2014 11:20:00 | 250.77 | 47.93 | 34.80 | 4.100 | 6.41 | 3.730 | 6.964 |
| 5/1/2014 11:25:00 | 269.25 | 47.54 | 35.55 | 4.192 | 6.50 | 3.780 | 7.067 |
| 5/1/2014 11:30:00 | 268.22 | 48.22 | 35.67 | 4.008 | 6.60 | 3.641 | 7.171 |
| 5/1/2014 11:35:00 | 376.16 | 48.50 | 35.45 | 5.394 | 6.69 | 4.911 | 7.274 |
| 5/1/2014 11:40:00 | 304.20 | 46.88 | 35.87 | 4.370 | 6.79 | 4.140 | 7.377 |
| 5/1/2014 11:45:00 | 304.20 | 47.50 | 35.73 | 4.435 | 6.88 | 4.198 | 7.48 |
| 5/1/2014 11:50:00 | 274.71 | 47.33 | 35.34 | 4.113 | 6.98 | 3.843 | 7.584 |
| 5/1/2014 11:55:00 | 291.37 | 48.36 | 35.30 | 4.483 | 7.07 | 4.180 | 7.687 |
| 5/1/2014 12:00:00 | 323.72 | 47.59 | 34.81 | 4.704 | 7.17 | 4.334 | 7.79 |
| 5/1/2014 12:05:00 | 303.02 | 47.62 | 34.30 | 4.514 | 7.26 | 4.216 | 7.893 |
| 5/1/2014 12:10:00 | 327.26 | 48.09 | 34.45 | 4.808 | 7.36 | 4.481 | 7.997 |
| 5/1/2014 12:15:00 | 385.56 | 49.19 | 36.53 | 5.164 | 7.45 | 4.659 | 8.1 |
| 5/1/2014 12:20:00 | 430.49 | 51.02 | 36.15 | 5.521 | 7.55 | 5.138 | 8.203 |
| 5/1/2014 12:25:00 | 196.44 | 46.28 | 33.68 | 2.879 | 7.64 | 2.743 | 8.307 |
| 5/1/2014 12:30:00 | 275.85 | 44.01 | 33.88 | 3.632 | 7.74 | 3.447 | 8.41 |
| 5/1/2014 12:35:00 | 341.05 | 44.33 | 35.57 | 4.847 | 7.83 | 4.496 | 8.513 |
| 5/1/2014 12:40:00 | 398.23 | 44.39 | 35.67 | 5.333 | 7.93 | 5.134 | 8.616 |
| 5/1/2014 12:45:00 | 598.43 | 44.60 | 37.85 | 7.018 | 7.97 | 6.789 | 8.667 |
| 5/1/2014 12:50:00 | 550.44 | 46.54 | 36.73 | 6.308 | 8.02 | 6.095 | 8.719 |
| 5/1/2014 12:55:00 | 517.22 | 46.13 | 37.11 | 5.913 | 8.07 | 5.631 | 8.77 |
| 5/1/2014 13:00:00 | 526.62 | 48.15 | 36.87 | 5.445 | 8.12 | 5.227 | 8.821 |
| 5/1/2014 13:05:00 | 505.72 | 48.21 | 36.19 | 5.316 | 8.16 | 5.100 | 8.872 |
| 5/1/2014 13:10:00 | 549.42 | 47.56 | 36.47 | 5.955 | 8.21 | 5.793 | 8.924 |
| 5/1/2014 13:15:00 | 422.89 | 49.07 | 37.22 | 4.832 | 8.26 | 4.677 | 8.975 |
| 5/1/2014 13:20:00 | 337.50 | 49.14 | 35.57 | 4.481 | 8.30 | 4.366 | 9.026 |
| 5/1/2014 13:25:00 | 280.03 | 49.47 | 36.07 | 4.033 | 8.35 | 3.900 | 9.077 |
| 5/1/2014 13:30:00 | 274.76 | 49.38 | 36.08 | 4.132 | 8.40 | 3.933 | 9.128 |
| 5/1/2014 13:35:00 | 299.61 | 49.29 | 36.37 | 4.319 | 8.45 | 4.089 | 9.18 |
| 5/1/2014 13:40:00 | 275.85 | 48.70 | 35.28 | 4.253 | 8.49 | 4.040 | 9.231 |
| 5/1/2014 13:45:00 | 275.85 | 46.54 | 35.71 | 4.151 | 8.54 | 3.910 | 9.282 |
| 5/1/2014 13:50:00 | 295.70 | 44.11 | 35.13 | 4.303 | 8.59 | 4.028 | 9.333 |
| 5/1/2014 13:55:00 | 275.85 | 43.43 | 35.11 | 4.242 | 8.63 | 3.973 | 9.385 |
| 5/1/2014 14:00:00 | 287.25 | 41.82 | 34.63 | 4.428 | 8.68 | 4.168 | 9.436 |
| 5/1/2014 14:05:00 | 279.27 | 40.74 | 33.87 | 4.362 | 8.73 | 4.057 | 9.487 |
| 5/1/2014 14:10:00 | 286.11 | 40.40 | 34.69 | 4.340 | 8.77 | 4.031 | 9.538 |
| 5/1/2014 14:15:00 | 327.20 | 42.31 | 36.67 | 4.449 | 8.82 | 4.193 | 9.589 |
| 5/1/2014 14:20:00 | 330.36 | 43.05 | 36.49 | 4.656 | 8.87 | 4.384 | 9.641 |
| 5/1/2014 14:25:00 | 515.13 | 45.11 | 37.44 | 6.534 | 8.92 | 6.340 | 9.692 |
| 5/1/2014 14:30:00 | 396.68 | 45.69 | 37.49 | 5.157 | 8.96 | 4.830 | 9.743 |
| 5/1/2014 14:35:00 | 380.12 | 45.83 | 36.37 | 5.298 | 9.01 | 4.921 | 9.794 |
| 5/1/2014 14:40:00 | 435.72 | 44.21 | 35.58 | 5.703 | 9.06 | 5.336 | 9.846 |
| 5/1/2014 14:45:00 | 408.76 | 44.87 | 37.29 | 5.323 | 9.11 | 5.108 | 9.897 |
| 5/1/2014 14:50:00 | 285.40 | 46.21 | 37.52 | 3.980 | 9.15 | 3.866 | 9.948 |
| 5/1/2014 14:55:00 | 261.03 | 44.09 | 35.33 | 3.871 | 9.20 | 3.717 | 9.999 |
| 5/1/2014 15:00:00 | 241.65 | 44.02 | 34.88 | 3.600 | 9.25 | 3.439 | 10.05 |
| 5/1/2014 15:05:00 | 256.74 | 41.88 | 34.07 | 3.880 | 9.29 | 3.693 | 10.102 |
| 5/1/2014 15:10:00 | 304.06 | 40.18 | 34.19 | 4.642 | 9.34 | 4.298 | 10.153 |
| 5/1/2014 15:15:00 | 267.07 | 40.90 | 35.87 | 4.216 | 9.39 | 3.942 | 10.204 |
| 5/1/2014 15:20:00 | 258.09 | 42.44 | 34.96 | 4.092 | 9.43 | 3.787 | 10.255 |
| 5/1/2014 15:25:00 | 276.94 | 44.08 | 35.22 | 4.291 | 9.48 | 3.934 | 10.307 |
| 5/1/2014 15:30:00 | 308.56 | 44.71 | 35.74 | 4.800 | 9.53 | 4.332 | 10.358 |
| 5/1/2014 15:35:00 | 268.22 | 46.12 | 35.98 | 4.127 | 9.58 | 3.749 | 10.409 |
| 5/1/2014 15:40:00 | 255.55 | 45.08 | 35.20 | 4.086 | 9.62 | 3.838 | 10.46 |
| 5/1/2014 15:45:00 | 269.31 | 41.78 | 34.77 | 4.271 | 9.67 | 3.920 | 10.511 |
| 5/1/2014 15:50:00 | 294.33 | 43.28 | 36.01 | 4.591 | 9.72 | 4.232 | 10.563 |
| 5/1/2014 15:55:00 | 279.12 | 44.77 | 36.73 | 4.276 | 9.76 | 3.914 | 10.614 |
| 5/1/2014 16:00:00 | 298.54 | 45.13 | 36.22 | 4.544 | 9.81 | 4.212 | 10.665 |
| 5/1/2014 16:05:00 | 328.09 | 45.75 | 37.65 | 4.891 | 9.86 | 4.664 | 10.716 |
| 5/1/2014 16:10:00 | 330.56 | 42.72 | 35.07 | 4.875 | 9.91 | 4.542 | 10.767 |
| 5/1/2014 16:15:00 | 410.35 | 44.00 | 37.37 | 5.584 | 9.95 | 5.281 | 10.819 |
| 5/1/2014 16:20:00 | 378.34 | 44.77 | 37.50 | 4.963 | 10.00 | 4.621 | 10.87 |
| 5/1/2014 16:25:00 | 323.36 | 44.92 | 35.86 | 4.700 | 10.05 | 4.485 | 10.921 |
| 5/1/2014 16:30:00 | 283.83 | 43.08 | 34.97 | 4.154 | 10.09 | 3.895 | 10.972 |
| 5/1/2014 16:35:00 | 255.79 | 41.18 | 34.03 | 3.810 | 10.14 | 3.635 | 11.024 |
| 5/1/2014 16:40:00 | 284.21 | 40.10 | 34.05 | 3.867 | 10.19 | 3.663 | 11.075 |
| 5/1/2014 16:45:00 | 196.26 | 39.69 | 33.61 | 2.937 | 10.24 | 2.733 | 11.126 |
| 5/1/2014 16:50:00 | 163.00 | 39.70 | 33.48 | 2.598 | 10.28 | 2.416 | 11.177 |
| 5/1/2014 16:55:00 | 149.27 | 39.99 | 33.24 | 2.402 | 10.33 | 2.269 | 11.228 |
| 5/1/2014 17:00:00 | 132.23 | 39.06 | 32.67 | 2.132 | 10.38 | 2.014 | 11.28 |
| 5/1/2014 17:05:00 | 122.11 | 38.78 | 32.53 | 1.913 | 10.42 | 1.844 | 11.331 |
| 5/1/2014 17:10:00 | 123.30 | 39.05 | 32.50 | 1.858 | 10.47 | 1.779 | 11.382 |
| 5/1/2014 17:15:00 | 117.41 | 37.91 | 31.90 | 1.770 | 10.52 | 1.691 | 11.433 |
| 5/1/2014 17:20:00 | 112.25 | 38.07 | 32.46 | 1.738 | 10.57 | 1.670 | 11.485 |
| 5/1/2014 17:25:00 | 119.42 | 38.49 | 32.80 | 1.733 | 10.61 | 1.606 | 11.536 |
| 5/1/2014 17:30:00 | 121.43 | 38.28 | 32.25 | 1.735 | 10.66 | 1.616 | 11.587 |
| 5/1/2014 17:35:00 | 115.35 | 37.67 | 31.88 | 1.672 | 10.71 | 1.555 | 11.638 |
| 5/1/2014 17:40:00 | 102.49 | 37.60 | 32.25 | 1.593 | 10.75 | 1.533 | 11.689 |
| 5/1/2014 17:45:00 | 94.34 | 37.77 | 32.36 | 1.408 | 10.80 | 1.366 | 11.741 |
| 5/1/2014 17:50:00 | 76.32 | 37.33 | 31.28 | 1.171 | 10.85 | 1.178 | 11.792 |
| 5/1/2014 17:55:00 | 75.23 | 37.02 | 31.33 | 0.984 | 10.90 | 1.015 | 11.843 |
| 5/1/2014 18:00:00 | 65.20 | 36.83 | 31.94 | 0.856 | 10.94 | 0.923 | 11.894 |
| 5/1/2014 18:05:00 | 56.75 | 36.99 | 31.61 | 0.777 | 10.99 | 0.844 | 11.946 |
| 5/1/2014 18:10:00 | 50.15 | 37.04 | 31.51 | 0.699 | 11.04 | 0.767 | 11.997 |
| 5/1/2014 18:15:00 | 50.15 | 36.75 | 31.90 | 0.578 | 11.08 | 0.617 | 12.048 |
| 5/1/2014 18:20:00 | 50.15 | 36.40 | 31.55 | 0.586 | 11.13 | 0.611 | 12.099 |
| 5/1/2014 18:25:00 | 51.29 | 36.21 | 31.50 | 0.631 | 11.18 | 0.652 | 12.15 |
| 5/1/2014 18:30:00 | 50.15 | 35.89 | 31.25 | 0.605 | 11.23 | 0.631 | 12.202 |
| 5/1/2014 18:35:00 | 50.15 | 35.64 | 30.92 | 0.528 | 11.27 | 0.561 | 12.253 |
| 5/1/2014 18:40:00 | 50.15 | 35.54 | 30.57 | 0.444 | 11.32 | 0.486 | 12.304 |
| 5/1/2014 18:45:00 | 42.27 | 35.50 | 30.39 | 0.300 | 11.37 | 0.352 | 12.355 |
| 5/1/2014 18:50:00 | 28.84 | 35.37 | 30.21 | 0.185 | 11.41 | 0.241 | 12.407 |
| 5/1/2014 18:55:00 | 25.08 | 35.30 | 30.04 | 0.081 | 11.46 | 0.119 | 12.458 |
| 5/1/2014 19:00:00 | 24.43 | 35.10 | 29.87 | 0.018 | 11.51 | 0.029 | 12.509 |
| 5/1/2014 19:05:00 | 25.08 | 34.82 | 29.51 | 0.000 | 11.56 | 0.000 | 12.56 |
| 5/1/2014 19:10:00 | 25.08 | 34.52 | 29.46 | 0.000 | 11.60 | 0.000 | 12.611 |
| 5/1/2014 19:15:00 | 22.37 | 34.18 | 29.35 | 0.000 | 11.65 | 0.000 | 12.663 |
